# The efficacy of long-lasting nets with declining physical integrity may be compromised in areas with high levels of pyrethroid resistance

**DOI:** 10.1186/1475-2875-12-368

**Published:** 2013-10-24

**Authors:** Eric O Ochomo, Nabie M Bayoh, Edward D Walker, Bernard O Abongo, Maurice O Ombok, Collins Ouma, Andrew K Githeko, John Vulule, Guiyun Yan, John E Gimnig

**Affiliations:** 1 KEMRI/CDC Research and Public Health Collaboration, PO Box 1578, Kisumu 40100, Kenya; 2 Department of Biomedical Science and Technology, School of Public Health and Community Development, Maseno University, Maseno, Kenya; 3 Department of Microbiology and molecular genetics, Michigan State University, East Lansing, MI, USA; 4 Centre for Global Health Research, Kenya Medical Research Institute, Kisumu, Kenya; 5 Department of Ecology & Evolutionary Biology, School of Biological Sciences, University of California, Irvine, CA, USA; 6 Division of Parasitic Diseases and Malaria, Centers for Disease Control and Prevention, Atlanta, GA, USA

## Abstract

**Background:**

Long-lasting insecticide-treated mosquito nets (LLINs) are a primary malaria prevention strategy in sub-Saharan Africa. However, emergence of insecticide resistance threatens the effectiveness of LLINs.

**Methods:**

Cross-sectional surveys of LLINs were conducted in houses of seven and four villages in Gem and Bungoma Districts in western Kenya, respectively. Condition (number and area of holes in the nets), number and species of mosquitoes resting inside them, and insecticidal activity of nets were quantified. Mosquitoes collected inside nets were allowed to lay eggs and progeny tested for susceptibility to deltamethrin and permethrin, pyrethoids commonly deployed in LLINs in western Kenya.

**Results:**

In Gem, 83.3% of nets were less than three years old and 32.4% had at least one hole of any size; while in Bungoma, 92% were less than three years old and 48% had at least one hole. No anopheline and five *Culex spp.* mosquitoes were found resting inside nets in Gem regardless of the number and size of holes, while 552 *Anopheles gambiae s.l.*, five *Anopheles funestus s.l.* and 137 *Culex spp.* were in nets in Bungoma*.* The number of mosquitoes resting inside nets increased with hole areas >50 cm in Bungoma. In WHO resistance assays, f1 offspring of samples collected in nets in Bungoma were 94 and 65% resistant to deltamethrin and permethrin, respectively. Nets from Bungoma retained strong activity against a susceptible laboratory strain, but not against f1 offspring of field-collected *An. gambiae s.s.* All *An. gambiae s.s.* samples collected in nets were homozygous for the *kdr* genotype L1014S.

**Conclusions:**

In areas with pyrethroid resistant vectors, LLINs with modest hole areas permit mosquito entry and feeding, providing little protection against the vectors. LLIN formulations develop large holes within three years of use, diminishing their presupposed lifetime effectiveness.

## Background

Insecticide-treated nets (ITNs) are an important tool to protect individuals against the morbidity and mortality caused by malaria [1-3]. The distribution of long-lasting insecticidal nets (LLINs) (factory-treated ITNs designed to retain insecticidal activity for up to three years [4]) by governments, non-governmental organizations (NGOs) and donors, has resulted in a dramatic increase in their ownership and contributed to the decline in malaria burden since 2000 [5]. Ownership and use of ITNs within households, as measured by the number of children under five years of age reported to have used an ITN the previous night, increased by three to tenfold between 2000 and 2008 in many African countries [5-7]. According to the latest World Malaria Report, 41% of children and 33% of all persons residing in malaria-endemic regions of sub-Saharan Africa reported sleeping under an ITN [5]. The distribution of LLINs in malaria endemic areas of Kenya has increased household ownership of any net to 70% and household ownership of an ITN to 60% [8]. The increase in ownership and use of ITNs has contributed to a significant decline in the burden of malaria throughout sub-Saharan Africa [5], including Kenya [9,10].

All mosquito nets act as a physical barrier, preventing access to vector mosquitoes and thus providing personal protection against malaria to the individual(s) using the nets [11]. However, untreated nets with even a few holes provide little protection [12]. The addition of pyrethroid insecticides serves to enhance the effectiveness of intact mosquito nets and to extend the effectiveness of nets with holes. The pyrethroids used to treat the ITNs have an exito-repellent effect, thus adding a chemical barrier to the physical one and enhancing personal protection by nets [11,13]. The insecticides incorporated in the ITNs kill malaria vectors that come into contact with them and when used by a majority of the target population, may provide protection for the entire community, including those who do not themselves sleep under an ITN [14-16]. A meta-analysis of data from trials of treated and untreated nets suggested that approximately half of the protection was derived from the physical barrier of the net and half from the chemical barrier [17].

In western Kenya, malaria transmission in the lowland areas around Lake Victoria has historically been very high, with entomological inoculation rates estimated to be as high as 300+ infectious bites per person per year [18-20]. However, the scaling up of ITNs and other malaria control tools has reduced transmission and malaria-associated morbidity and mortality. Between 2003 and 2007, demographic surveillance indicated a 42% reduction in all-cause mortality among children less than five years of age coinciding with a scale up of ITNs as well as improvement in diagnostics and introduction of ACT [21]. *Anopheles gambiae s.l.* and *Anopheles funestus* densities declined markedly in a randomized trial of permethrin-treated bed nets in a comparison of treatment *versus* control villages in western Kenya [22,23]. Subsequent monitoring demonstrated a decline in the proportion of *An. gambiae* s.s.*,* the principal vector of malaria, relative to *Anopheles arabiensis*[24].

However, a resurgence in malaria vectors, parasite prevalence and malarial disease burden has been observed in several sites in western Kenya despite high ownership of ITNs [25,26]. This resurgence could be due to one or a combination of the following factors: reduced efficacy of ITNs, insecticide resistance in mosquitoes, improper use of ITNs, stock-outs of anti-malarial drugs or even a poor dosing regimen of policy recommended drugs by private outlets [25,27]. This study investigated the impact of high levels of pyrethroid resistance in *An. gambiae* on their tendency to enter and rest inside nets. The results suggest that pyrethroid resistance in mosquitoes may undermine the effectiveness of nets with even a few holes. These findings have serious implications for malaria control programmes in sub-Saharan Africa where resistance to pyrethroid insecticides is increasing rapidly [28].

## Methods

### Study sites

Bed net surveys were conducted in seven villages in Gem district (0°07′30.06″N, 34°24′32.56″E) in Siaya County and four villages in Bungoma district (0°35′20.85″N, 34°29′13.23″ E) in the month of May 2013. The two study sites are about 100 km apart. Inhabitants of Bungoma include subsistence and large-scale farmers growing cash crops such as sugar cane, tobacco, onions, and tomatoes. Residents of Gem are primarily subsistence farmers growing food crops. The ITN coverage in the two sites is 80 and 70% for Gem and Bungoma, respectively [10,29].

Malaria transmission in both areas is high. The main malaria vectors are *An. gambiae s.s.*, *An. arabiensis* and *Anopheles funestus*. Previous studies have shown high resistance to pyrethroids [30] in *An. gambiae* in both areas. The species composition of these areas differs. *Anopheles gambiae* comprises >70% of the *An. gambiae s.l.* mosquitoes collected from houses in Bungoma, whereas *An. arabiensis* accounted for up to 90% of the *An. gambiae s.l.* in Gem and surrounding areas [24].

### Bed net cross-sectional survey

Community-based, cross-sectional surveys were carried out in the two study sites. Sampling was done in all the houses in the selected villages in Bungoma and in randomly selected houses in the selected villages in Gem. The surveys consisted of interviews with household heads and an inspection of all nets in the house. Interviews were conducted using a structured questionnaire administered on personal data assistants (PDAs, Dell Axim X50, Dell Inc, Dallas, TX, USA) and collected information on the household characteristics: wall type, roof type, whether eaves were open or closed, insecticide use and application within the household, frequency of net use, the number of people who used the nets the previous night and the brands and ages of the nets. The interviewers examined the nets for the presence of mosquitoes resting inside them using torches. Any mosquitoes observed were collected using a mouth aspirator, transferred into a paper cup and labeled with the house ID and net type. The samples were placed in a cool box and maintained on a 10% sugar solution for transport to insectary. The interviewers then examined each net and recorded the presence, number and size of holes. Hole sizes were categorized using methods recommended by the WHO Pesticide Evaluation Scheme: the thumb was used to estimate hole sizes ≤2 cm in diameter (small), holes larger than the thumb but smaller than the fist were estimated to be between 2 and 10 cm (medium), while those larger than the fist were estimated to be ≥10 cm (large). Holes that fell within the largest hole size category (>25 cm) recommended by WHOPES [31] were recorded as large holes (≥10 cm).

### Mosquito rearing

Female mosquitoes were sorted based on species (*An. gambiae s.l.* and *An. funestus s.l.*) and on abdominal status. Fed and half-gravid samples collected from the nets were maintained on 10% sugar solution at the KEMRI/CDC insectary until they became gravid. Gravid mosquitoes were placed in oviposition cups containing laying pads made of moist cotton wool covered with filter paper. Mosquitoes were pooled into 34 oviposition cups with one to five females per cup. To ensure maximum hatch rate, once the females laid eggs, the laying pads with eggs were transferred to a hatching bowl. Hatched larvae from each cup were transferred to a larval tray. Larvae were fed daily on a mixture of brewer’s yeast and fish food, and their water changed every two days. Pupae from the same larval tray were transferred to the same eclosion cup and placed inside individual paper cups for emergence. Freshly emerged adults were fed on 5% sugar solution for three days after which they were ready for bioassays. In addition, mosquitoes were collected as larvae from each site and reared until three days old adults as above.

### Susceptibility testing

To assess susceptibility to insecticides, field collected mosquitoes (three days old) were exposed to permethrin and deltamethrin using WHO tube tests [32]. Three sets of samples were exposed: f1 offspring of the *Anopheles* samples collected inside mosquito nets from Bungoma and adult samples from larval collections in Bungoma and Gem.

### Bed net bioassays

In Bungoma, 68 of the nets with mosquitoes resting inside and 31 nets without were collected from the field and the owners provided with new LLINs in exchange. WHO cone bioassays using five to ten mosquitoes on five pieces cut from these nets, one piece per side and one from the top [33] were performed using the susceptible *An. gambiae s.s.* Kisumu strain. In addition, the progeny of females collected from inside nets were exposed to new, unused, unwashed PermaNet 2.0 (Vestergaard Frandsen SA, Aarhus, Denmark) and Olyset (Sumitomo Chemicals, Osaka, Japan) as these were the primary net brands observed in the field. For all bioassays, mosquitoes were exposed in plastic cones for 3 min and then transferred to holding cages with access to 10% sucrose solution. Knockdown was recorded 60 min after exposure and mortality was recorded 24 hours after exposure.

### Molecular assays

Conventional polymerase chain reaction (PCR) was used to distinguish between the two sibling species of the *An. gambiae* species complex native to western Kenya, *An. gambiae s.s.* and *An. arabiensis*[34]. Further, the mosquito samples were tested for the presence of the 1014S *kdr* genotype using the methods previously described [35] and as modified in Mathias *et al.*[36]. Sporozoite ELISA was also conducted on the head and thorax of all the female mosquitoes [37].

### Data analysis

The average hole area was estimated according to the methods recommended by the WHO Pesticide Evaluation Scheme [33]. The data are presented in square centimetres instead of as a proportionate hole index. A Poisson regression model, corrected for over-dispersion, was used to estimate the effect of net type, net age and the physical condition of the net, and the number of mosquitoes resting inside. The hole area was categorized as ≤50 cm^2^, 50–500 cm^2^ and >500 cm^2^. Nets with no holes were used as the reference category in the regression analysis.

### Ethical clearance

Ethical clearance was obtained from the Ethical Review Committee of the Kenya Medical Research Institute (SSC 2267) and the Institutional Review Boards of the US Centers for Disease Control and Prevention (IRB 6395).

## Results

### Bed net cross-sectional survey

The bed net survey was conducted in a total of 482 households: 303 in Bungoma and 179 in Gem, with a total of 590 nets being sampled. The numbers of the different types of nets and their ages are shown in Table 1. In Gem, just over half (117/216, 54.0%) of the nets were PermaNets while 69/216 (32.1%) were Olyset nets. In Bungoma, most of the nets were Olyset nets (279/374, 74.7%) while only 58/374 (15.6%) were PermaNets. The remainder of the nets sampled in both sites (57/590, 6.7%) were SupaNets [commercially available, conventionally treated nets bundled with deltamethrin treatment kits (KO-Tab®)] or nets that could not be identified. In both sites, most nets were less than three years old; 84.5% were less than or equal to three years old in Gem and 91.6% were less than or equal to three years old in Bungoma. The percentage of nets with at least one hole was 32.4% in Gem and 48% in Bungoma. Five *Culex spp.* mosquitoes and no anophelines were collected from inside nets in Gem while in Bungoma, a total of 552 *An. gambiae s.l.*, five *An. funestus s.l.* and 137 *Culex spp.* were collected inside nets*.* Hereafter, results are reported only for Bungoma.

**Table 1:** Number of each type of bed net in the two study sites

**Type of ITN**	**Gem**			**Bungoma**		
	**≤ 3 years**	**> 3 years**	**Unknown**	**≤ 3 years**	**>3 years**	**Unknown**
Olyset	59 (85.5)	10 (14.5)	0 (0.0)	254 (91.7)	14 (5.1)	9 (3.3)
Permanet 2.0	96 (82.1)	20 (17.1)	1 (0.9)	48 (78.7)	8 (13.1)	5 (8.2)
Supanet*	1 (33.3)	2 (66.7)	0 (0.0)	27 (75.0)	8 (22.2)	1 (2.8)
Other	24 (88.9)	3 (11.1)	0 (0.0)	0	0	0
**Total**	**180 (83.3)**	**35 (16.2)**	**1 (0.5)**	**329 (88.0)**	**30 (8.0)**	**15 (4.0)**

Numbers in parentheses indicate the percent of each net type that are less or equal to 3 or than or greater than three years of age as reported by the owner in each site.

*A conventional net available at local shops.

Overall, 36% of nets in Bungoma had at least one *Anopheles* mosquito resting inside the net. Among the different net types, 38% of the Olyset nets, 33% of the Permanet 2.0 nets and 29% of all Supanet nets had at least one *An. gambiae s.l.* resting inside. The proportion of nets less than or equal to three years old and those older than three years that had at least one mosquito resting inside were 36.9 and 36.6%, respectively. The proportion of intact nets (without holes) and damaged nets (with one or more holes) that had at least one mosquito resting inside were 37 and 48%, respectively.

The mean number of *Anopheles* mosquitoes found within nets increased in nets with hole sizes above 50 cm^2^ (Table 2). The number of mosquitoes collected from nets with total hole areas less than 50 cm^2^ was not significantly different from nets with no holes. For nets with hole areas 50–500 cm^2^ and >500 cm^2^, there were generally twice as many mosquitoes observed resting inside compared to nets with no holes (RR = 2.08, *P* = 0.001 for 50–500 cm^2^; RR = 1.89, *P* = 0.012 for >500 cm^2^) (Table 3). There was no increase in the number of mosquitoes found resting in nets with hole areas >500 cm^2^ compared to nets with hole areas between 50–500 cm^2^ suggesting a threshold effect. There was no significant difference in the number of mosquitoes found resting in the different net types, nor in nets in different age categories (Table 3).

**Table 2:** Mean number of mosquitoes found in nets in Bungoma by the hole area

**Hole index category**	**Sample size**	**Mean number of *Anopheles *** **per net**	**Lower 95% Confidence intervals**	**Upper 95% Confidence intervals**	**Nets with Mosquitoes inside**
0 cm^2^	163	1.01	0.67	1.34	37%
<50 cm^2^	55	1.40	0.73	2.07	24%
50-500 cm^2^	103	2.06	1.3	2.78	41%
>500 cm^2^	53	1.91	0.94	2.87	40%

**Table 3:** **Results of a Poisson regression model of the number of *An. gambiae *** **s.l. inside ITNs in Bungoma County including the model estimates, risk ratios and P-values**

**Parameter**	**Risk Ratio**	**P-value**
**Net type**		
Olyset	0.85 (0.50, 1.46)	0.563
PermaNet	0.74 (0.38-1.47)	0.394
SupaNet	Ref.	Ref.
Net age*		
Less than 3 years	1.02 (0.56-1.84)	0.945
Older than 3 years	Ref.	Ref.
**Hole index category**		
>500 cm^2^	1.89 (1.148-3.111)	**0.012**
50 to 500 cm^2^	2.08 (1.38-3.12)	**<0.001**
<50 cm^2^	1.34 (0.8-2.36)	0.243
No holes	Ref.	Ref.

The 95% CI for the estimate and risk ratios are given in parentheses. Significant *P*-values are given in boldface.

*Nets of unknown age were categorized as older than three years. Additional models were run with nets of unknown aged categorized as less than or equal to three years and the results did not change substantially.

### Susceptibility of mosquito samples to insecticides

**Figure 1:**
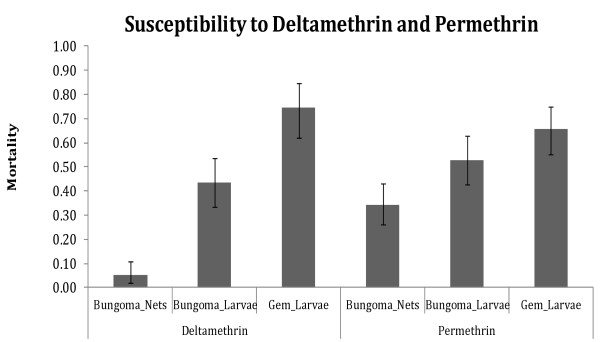
**Susceptibility status of mosquito populations.** Mortality of *An. gambiae s.l.* mosquito samples when exposed to deltamethrin and permethrin. Bungoma_Nets represents mortality of f1 offspring of mosquitoes collected resting inside nets in Bungoma, Bungoma_larvae represents mortality of samples collected as larvae in Bungoma and reared to adults for exposure while Gem_larvae mortality represents mortality of samples collected as larvae in Gem and reared to adults for exposure.

Within Bungoma, mosquitoes reared from females collected inside nets had lower susceptibility (5% to deltamethrin and 34% to permethrin) compared to those from larval collections (43% to deltamethrin and 53% to permethrin). Mosquitoes from Bungoma, whether collected from inside nets or as larvae generally showed lower susceptibility compared to mosquitoes collected as larvae from Gem (75% to deltamethrin and 65% to permethrin). In Gem, there were no mosquitoes collected from inside nets (Figure 1). Based on the current WHO insecticide susceptibility guidelines, populations from Bungoma and Gem would be classified as resistant to the two insecticides [32].

### Mosquito bioassays

Bed net bioassays were conducted on a sample of the field-collected nets with mosquitoes present inside (N = 68) and compared with those in which no mosquitoes were found (N = 32). Average knockdown and mortality were above 90% for nets that had mosquitoes present inside and for those in which no mosquitoes were observed resting inside on the day of collection (Table 4). No significant differences were observed in the knockdown or mortality of the susceptible strain to the two groups of nets (Table 4). When exposed to brand new, unused, unwashed nets, *An. gambiae s.s.* from Bungoma had 57.5% knockdown and 37.5% mortality to Olyset (N = 40) and 54% knockdown and 22% mortality (N = 50) to PermaNet 2.0 nets.

**Table 4:** **Mean knockdown and mortality of susceptible *Anopheles gambiae *** **s.s. Kisumu strain when exposed to field collected nets with mosquitoes present (N = 68) or absent (N = 32) at the time of collection and the comparison of knockdown and mortality by logistic regression**

**Outcome**	**Mosquitoes**	**Mean**	**Odds Ratio**	**P-value**
Knockdown	Present	94.4	1.20 (0.88, 1.80)	0.201
	Absent	92.9	Ref.	
Mortality	Present	92.5	0.74 (0.51, 1.06)	0.108
	Absent	94.2	Ref.	

The 95% CI for the estimate is given in parentheses.

### Molecular assays

All the mosquitoes collected inside nets were identified as *An gambiae s.s.* (N = 283), while 40 samples did not amplify and were not identified. Among mosquitoes collected by pyrethrum spray collections (PSC), 73% were *An. gambiae s.s.* and 27% were *An. arabiensis* (N = 88) while 12 samples did not amplify. All the *An. gambiae s.s.* samples collected from inside the nets were homozygous for *kdr* 1014S (N = 343) and 1.8% of the *An. gambiae s.s.* collected inside nets tested positive for *Plasmodium falciparum* sporozoites (N = 343). A total of 95.8% of the samples collected from PSCs in Gem were *An. arabiensis* with only 4.2% being *An. gambiae s.s.* (N = 48).

## Discussion

In Bungoma, an area with high levels of pyrethroid resistance [30,36], live *Anopheles* mosquitoes were routinely observed resting inside nets. This may have been due to declining bioefficacy of the nets, reduced susceptibility of the mosquitoes to pyrethroid insecticides or both. Susceptibility testing using females reared from field-collected larvae or from the f1 generation derived from mosquitoes collected inside nets confirmed high resistance to pyrethroids. Average knockdown and mortality of a susceptible strain of mosquitoes exposed to field-collected nets was greater than 90% regardless of whether the mosquitoes had been observed resting inside, indicating that the nets in the field generally had adequate levels of insecticide: the average mortality rates were well above the 80% threshold recommended by WHOPES as a criteria for a functional ITN/LLIN [31]. In contrast, the mortality of f1 adult mosquitoes raised from adult mosquitoes collected inside nets in Bungoma and exposed to unused, unwashed Olyset and PermaNet 2.0 nets was 37.5 and 22%, respectively. Several mosquitoes collected inside the nets were positive for *P. falciparum* sporozoite infection.

In contrast to Bungoma, no mosquitoes were observed inside 213 nets in Gem. Although no formal analysis was done, there were several differences between the two sites. The main difference was that the *Anopheles* population in Gem is comprised largely of *An. arabiensis.* This species has lower resistance to pyrethroid insecticides [30,36], which may have reduced the likelihood that mosquitoes would enter an ITN and survive exposure. This species is also more likely to feed on alternative hosts compared to *An. gambiae s.s.*, which may allow it to persist in presence of high coverage of ITNs [24]. *Anopheles gambiae s.s.* historically was very common in Gem and surrounding areas and it is still present at low levels. It has been shown to be resistant to pyrethroid insecticides (unpublished data) and it is not clear why its numbers have not rebounded or why none were observed resting inside nets. It is possible that the resistance mechanism or intensity of resistance in *An. gambiae* in Gem is different from that of Bungoma, where mass LLIN campaigns were first conducted in 2011. Alternatively, there may be additional ecological constraints, which in combination with the widespread use of ITNs, result in the continued suppression of the population of *An. gambiae s.s.* in Gem.

Pyrethroid resistance has been spreading rapidly in sub-Saharan Africa and has been documented in 23 countries [28]. This may partly be in response to agricultural application and run-off of insecticides into mosquito breeding sites [38-40], but increasingly in response to selection pressure resulting from the scale up of insecticide-treated nets and indoor residual spraying as malaria prevention tools [4,36,41-45]. Regardless of the source of insecticide pressure, insecticide resistance in malaria vectors has been predicted to eventually undermine control programmes that are solely reliant on insecticides such as indoor residual spraying (IRS) and ITN programmes [28]. While pyrethroid resistance has been documented in malaria vectors throughout sub-Saharan Africa, there is surprisingly little information on the impact of resistance on the effectiveness of vector control efforts. An experimental, hut trial in two sites in Benin, one with susceptible mosquitoes and the other with resistance to pyrethroids, showed blood-feeding was reduced by 96% at the site with susceptible vector population, but was largely unaffected at the site with high levels of pyrethroid resistance, while the mortality of mosquitoes entering huts at the susceptible site was nearly three times as high as that at the site with high levels of pyrethroid resistance [46]. Household trials in other parts of Benin also showed that sleeping under an ITN in an area with resistant mosquitoes was no more protective than sleeping under an untreated net, regardless of its physical condition [47]. During a longitudinal study of inhabitants of Dielmo village, Senegal, a rise in the incidence of malaria following the distribution of LLINs was attributed to increasing pyrethroid resistance in the local vector populations [48]. In contrast, a study in Ivory Coast found no reduction in the protective efficacy of ITNs in an area with high levels of pyrethroid resistance [41], while in Malawi, increasing pyrethroid resistance in *An. funestus* was not associated with an increase in malaria transmission in areas with LLINs although in areas with IRS, no additional impact was observed [49]. In Benin, mosquitoes were collected from inside nets with 12 holes that were 4 cm × 4 cm. Insecticide treated nets reduced the number of mosquitoes entering compared to an untreated net but an average of 5 mosquitoes were collected each night under LLINs [50]. A modeling study to measure the effect of pyrethroid resistance on the cost effectiveness of LLINs showed strong, positive correlations between insecticide susceptibility status and predicted population level insecticidal effectiveness of and protection against blood feeding by LLIN intervention programmes [51]. With the most resistant mosquito population, LLIN mass distributions would avert up to 40% fewer episodes of malaria compared to areas with a fully susceptible population [51]. An ongoing study in western Kenya shows prospects of generating evidence within the next year or two on the impact of insecticide resistance on the efficiency of malaria control interventions (Mbogo, pers. comm).

Several factors associated with the number of mosquitoes inside nets were explored. As described above, the location was a strong determinant of the presence of mosquitoes inside nets, presumably due to the composition of the local vector population, and further analyses included only Bungoma. In that site, neither net brand nor the age of the nets was associated with the number of mosquitoes inside nets. Although the nets were not stratified by age, the study demonstrated high mortalities of susceptible mosquitoes exposed to nets collected from the field indicating that most nets had adequate levels of insecticide. An increase in the number of mosquitoes inside nets with increasing levels of physical damage was however, observed. Nets with estimated hole areas of >50 cm^2^ had more mosquitoes than nets with no holes. Although the sample sizes were limited, the data suggested that a threshold is reached beyond which increasing damage does not lead to increasing numbers of mosquitoes. This may indicate that beyond a certain amount of damage, nets are equally likely to be penetrated by mosquitoes. However, the possibility that increasing damage also allows for more mosquitoes to escape from nets, which may also account for the apparent threshold effect, cannot be ruled out. Interestingly, nets with no holes had an average of just over one mosquito per net. Presumably, this was due to improper usage and residents should be instructed on how to tuck their nets in to prevent mosquitoes from entering them.

It has been suggested that the physical integrity of the LLINs may be compromised before the insecticidal activity falls below established thresholds indicating the need for replacement [49,50] and multiple reports have documented physical damage to nets under conditions of routine use. Rehman *et al.* noted that 39, 24 and 63% of all the nets in use in Bioko Island, continental Equatorial Guinea and Malawi, respectively, were holed within two years of distribution [52]. During a long-term assessment of a polyester-based LLIN in Uganda, more than 70% of nets had holes after only one year and more than 85% after two years [53]. Wills *et al.*, reported 54.5% of nets having holes after just six months of distribution in Ethiopia [54]. In Kenya, in an ongoing net durability study in western Kenya, it was observed that up to 40% of some net types had holes within six months of deployment in Siaya County (Bayoh, pers. comm.) while some recent surveys reported that up to 74% of the bed nets in use in Kwale County had holes [55,56]. The WHOPES guidelines on monitoring the durability of LLINs outline methods to estimate the hole sizes on the net fabric [31]. However, the guidelines do not provide criteria for physical damage that is indicative of net failure and requiring the replacement of the nets. Mutuku *et al.* proposed a pHI of 88 corresponding to approximately 500 cm^2^ of damage [55] while Gnanguenon *et al.* observed mosquitoes entering nets with 12 holes 4 cm × 4 cm corresponding to a proportionate hole indexes (pHI) of 276 [50,55]. Several authors have suggested criteria based upon pHI and the probability that owners will discontinue use due to the owners’ perception that the nets are no longer effective [57,58]. The cut-off for an unacceptable net ranged from a pHI of 300 in Chad corresponding to a hole area of approximately 1,000 cm^2^ to a pHI of 764 in Ethiopia which corresponded to a hole area of approximately 1,200 cm^2^. In studies in Bioko Island and Malawi, the risk of malaria increased with deteriorating condition of nets with untreated nets with at least one hole providing the least protection [52] although specific thresholds for net replacement were not presented. The data suggest that in areas with high levels of pyrethroid resistance, the threshold for a net requiring replacement may be at the lower end of the spectrum. While there is complex relationship between hole area and insecticidal activity of the nets and insecticide resistance and behaviour of the vector population, specific criteria for the physical integrity of nets should be developed to assist national malaria control programmes in determining the appropriate replacement strategies for LLINs.

The spread of pyrethroid resistance combined with increasing evidence that it may compromise malaria vector control programmes highlights the need for new insecticides and new tools for malaria prevention. Currently, LLINs are treated with pyrethroid insecticides only and their loss as an effective tool would seriously undermine malaria control programmes throughout sub-Saharan Africa. IRS with non-pyrethroid insecticides is an option that is immediately available. However, IRS is expensive relative to LLINs, particularly when spraying is done with non-pyrethroids, and is unlikely to be widely implemented without a significant increase in the amount of funding available for malaria control programmes. Two new LLIN products are currently available that incorporate a synergist to mitigate the effects of pyrethroid resistance. The Permanet 3.0 is treated with deltamethrin on the sides and deltamethrin plus piperonyl butoxide (PBO) on top. PBO is a synergist and increases the potency of the pyrethroid insecticides by inhibiting oxidase enzymes that have been implicated as one mechanism of resistance [59]. Evidence that the PermaNet 3.0 is more effective than the PermaNet 2.0 which is treated with deltamethrin alone, however, is limited and occasionally mixed, presumably due to the presence of other resistance mechanisms that are unaffected by PBO and the WHO Pesticide Evaluation Scheme did not recommend this product for use as a resistance management tool [60]. The Olyset Plus is another bi-treated net with permethrin plus PBO throughout the net [33]. However, there is limited data on the efficacy of this net against wild populations of mosquitoes that are resistant to pyrethroid insecticides. Larviciding is an alternative vector control tool with a wide range of activity that are recommended for use against malaria vectors. However, as with IRS, larviciding can be expensive and is currently only recommended for specific settings. Other insecticides such as chlorfenapyr, indoxacarb [46], and diafenthiuron [61] are being investigated as options for IRS but it is likely to be several years before commercially available formulations will be available. Spatial repellents [62] and toxic sugar baits [63] have also been proposed for malaria prevention but these too require several years of evaluation and refinement before they can be considered viable tools for malaria control programmes.

While this study has demonstrated that pyrethroid resistant mosquitoes are entering and surviving exposure to LLINs, the results should not be interpreted to indicate that LLINs are no longer useful in malaria control programmes. First, while pyrethroid resistance is widespread, the intensity of resistance in many areas is likely low and in these areas LLINs may still be effective. The lack of mosquitoes collected inside nets in Gem demonstrates that LLINs are not compromised everywhere and differences in the intensity of resistance, as well as the effectiveness of LLINs, may vary over relatively short distances. Second, this study was a cross-sectional survey and differences in the age of mosquitoes may affect their susceptibility to pyrethroid insecticides as older mosquitoes have been shown to be more susceptible. Additionally, mosquitoes may repeatedly encounter insecticides over their life and, although this has not been demonstrated, the cumulative exposure may eventually result in the death of the mosquitoes. However, the finding of *P. falciparum*-infected mosquitoes inside nets suggests that, at least in Bungoma, older mosquitoes are able to survive exposure to treated nets. Lastly, intact untreated nets still provide some protection and there may be community-wide effects where malaria transmission is reduced when most people in the population regularly sleep under nets. Without baseline data on the effectiveness of LLINs before the rise of pyrethroid resistance, the impact of resistance on the effectiveness of LLINs cannot be reliably measured. However, the data strongly suggest that the efficacy of pyrethroid-treated nets may be compromised in areas with high levels of pyrethroid resistance.

## Conclusion

The study found *An. gambiae* mosquitoes resting inside pyrethroid-treated nets in an area of documented pyrethroid resistance in western Kenya. It was confirmed that the local mosquitoes were resistant to permethrin and deltamethrin, that they survived exposure to unused, unwashed LLINs in standard cone bioassays and that field-collected nets retained adequate levels of insecticide as measured in bioassays against a susceptible strain of *An. gambiae* mosquitoes. The data presented here indicate that resistance in *Anopheles* mosquitoes in Bungoma may be undermining the efficacy of the nets in the area. It was demonstrated that nets with holes were more likely to harbour resting mosquitoes. Given the spread of pyrethroid resistance throughout sub-Saharan Africa, it is hoped that other researchers will adopt this methods to examine how well bed nets are preventing mosquito entry in other sites. These findings highlight the need for new insecticides and tools for malaria prevention in Africa as well as the need to refine LLIN replacement strategies. Failure to address the spread of pyrethroid resistance threatens to undermine the gains made in the prevention and control of malaria in sub-Saharan Africa.

## Competing interests

The authors declare that they have no competing interests.

## Authors’ contributions

EO, JEG, NMB, MO, BA, CO, AK, JV, YG and EDW designed the study, developed the study and took part in the manuscript preparation. EO, JEG and NMB contributed to development of the protocol and data analysis. BA and EO performed the laboratory analysis of the samples. All authors read and approved the final manuscript.
